# Adjunctive Serum Drops to Paper Patch for Tympanic Membrane Perforation

**DOI:** 10.1177/00034894261436880

**Published:** 2026-04-06

**Authors:** Yalda Yazdani, Ella J. Lee, Karen Tawk, Mehdi Abouzari, Hamid R. Djalilian

**Affiliations:** 1 Department of Otolaryngology-Head and Neck Surgery, University of California, Irvine, CA, USA; 2 Department of Neurological Surgery, University of California, Irvine, CA, USA; 3 Department of Biomedical Engineering, University of California, Irvine, CA, USA

**Keywords:** myringoplasty, paper patch, perforation, serum drops, tympanic membrane

## Abstract

**Objective:**

To evaluate the effectiveness of autologous serum drops combined with paper patch myringoplasty in enhancing tympanic membrane (TM) perforation closure among patients with prior patch failure.

**Methods:**

This retrospective chart review included 81 patients with chronic TM perforations unresponsive to initial paper patching. Patients were divided into 2 groups: the experimental group received autologous serum drops alongside secondary paper patching, while the control group received patching alone. The primary outcome was TM closure at 8 weeks. Kaplan–Meier survival analysis and Cox regression were used to evaluate time-to-closure and the effect of patient variables.

**Results:**

Closure rates were significantly higher in the experimental group (54.5%) compared to the control group (21.6%; *P* = .002). The addition of serum drops was associated with improved healing outcomes, even in cases unresponsive to initial patching. Perforation size and location influenced closure, with smaller and posterior TM perforations showing higher success. No significant associations were found between healing and age, sex, race, comorbidities, or perforation etiology. No adverse effects from serum drops were reported.

**Conclusion:**

Autologous serum drops significantly improve TM closure rates when added to paper patch myringoplasty, offering a safe, cost-effective, and non-surgical option for managing chronic perforations. Further studies with larger sample sizes and long-term follow-ups are warranted.

## Introduction

Tympanic membrane (TM) perforations are common and can result in conductive hearing loss and recurrent infections. They typically arise from otitis media, traumatic injury, barotrauma, or iatrogenic causes such as ventilation tube placement.^1,2^ Chronic OM is the most common etiology, particularly in developing regions where untreated infections contribute significantly to TM damage.^3^ Acute traumatic TM perforations, especially small ones, have the highest rate of spontaneous healing based on their etiology, duration, and size.^4^ However, larger perforations from chronic infection show the lowest spontaneous healing rates and worse clinical outcomes.^4,5^ If left untreated, these can cause conductive hearing loss, recurrent ear infections, and cholesteatomas. Thus, nonhealing perforations usually need intervention to restore TM integrity, improve hearing, and prevent complications.

Tympanoplasty and myringoplasty are standard surgical treatments for TM perforations but involve challenges like the need for an operating room, general anesthesia, and additional costs.^6^ Several alternative methods have been explored as substitutes for traditional myringoplasty and tympanoplasty, including paper patches and chitin membranes.^7,8^ These methods are simple, cost-effective, and can be performed non-surgically in the office.^8^ These techniques showed considerable efficacy. For instance, paper patching achieved closure rates of 63.2%, 43.5%, and 12.5% for small, medium, and large perforations, respectively.^9^ Another myringoplasty involves the adjunctive application of regulatory factors crucial for wound healing. Studies showed that hyaluronic acid, epithelial growth factor, and fibroblast growth factor aid TM repair with positive outcomes.^10-18^ A natural source of these bioactive substances is autologous blood serum,^17,18^ which has demonstrated effectiveness in promoting healing across various types of wounds, including corneal wounds.^19-21^ However, previous research on the use of autologous blood serum for TM healing has yielded mixed results or has been limited by unclear factor correlations.^17^ This study assessed the effectiveness of autologous serum drops with paper patching for TM perforation closure in patients with prior patch failures.

## Materials and Methods

### Participants

Following Institutional Review Board approval, we performed a retrospective chart review of patients who visited our tertiary care neurotology clinic from 2018 to 2022. We included patients with TM perforations lasting more than 6 months, classified as chronic, and resulting from various etiologies. Patients with prior middle ear surgery, head and neck radiotherapy, or nasopharyngeal and skull base issues causing eustachian tube dysfunction were excluded due to reduced efficacy and lower success rates of TM closure. Additionally, perforations occurring within 6 months were excluded because they have a high likelihood of spontaneous healing. Including these cases could lead to an overestimation of the intervention’s success rate due to the natural healing process. All patients underwent primary paper patch placement, with no size change observed on microscopy during the 8-week follow-up period.

### Study Design and Techniques

This chart review study assessed the efficacy of autologous serum drops with paper patching for TM perforation closure compared to paper patch alone in patients who had previously failed a paper patch procedure. After failure of the primary patch, patients were counseled regarding management options. Group allocation was based on patient preference, resulting in 2 groups: an intervention group receiving secondary paper patching with autologous serum drops, and a control group receiving secondary patching without serum drops. The secondary paper patching procedure was performed similarly to the primary paper patch. This process involved treating the edges of the perforations with 25% trichloroacetic acid, refreshing the edges with a hook, and then placing a sterile paper patch before applying a drop of ofloxacin 0.3% ophthalmic. The procedure was performed in an outpatient setting using microscopic visualization. In the experimental group, written informed consent was obtained prior to blood collection. Four tubes of the patient’s venous blood were obtained and centrifuged at 3000 RPM for 15 minutes, and the serum was mixed with 5 cc of ofloxacin 0.3% ophthalmic drops. Three drops were applied to the paper at the end of patch placement. The patient was asked to keep the drops refrigerated and use them 3 drops twice daily for 6 weeks. The control group received the same patching procedure but without serum.

### Follow-Up and Outcome Assessment

We analyzed outcomes over 8 weeks, until TM perforation closure occurred or the time limit was reached. Closure rates were assessed through microscopic examination and tympanometry. The primary outcome was complete TM closure; the secondary outcome was reported adverse effects related to the serum drops application.

### Statistical Analysis

Survival analysis was conducted to evaluate TM perforation closure rates over an 8-week follow-up period. Time to closure was measured in weeks, with censoring applied to patients who did not achieve TM closure within the study duration. Kaplan-Meier survival analysis was first used to compare cumulative closure rates between the experimental and control groups, with statistical differences assessed using the log-rank test. In order to avoid violating the structure of standard survival modeling, we defined a single healing event endpoint (no closure vs complete closure). Following this, Kaplan-Meier analysis was also applied within the experimental group to examine the impact of categorical variables on TM closure rates, further evaluating differences using the log-rank test. For the analysis of continuous variables, in particular age, a Cox proportional hazards model was employed to estimate hazard ratios (HRs) and 95% confidence intervals (CIs), assessing its influence on TM perforation closure. All statistical analyses were conducted using R version 4.4.1 in Posit Cloud (Posit Software, Boston). The ggsurvfit and survival packages were used for Kaplan-Meier and Cox regression analyses.

## Results

A total of 81 patients (81 perforated TMs) were included in the study, with a mean age of 58 ± 20 years. Of these, 37 (46%) were in the control (paper alone) group and 44 (54%) were in the experimental (paper and serum) group. The cohort comprised 37 (54%) female and 44 (46%) male patients. The 2 groups had no significant differences in demographic and baseline characteristics before treatment (Table 1). In the experimental group, 18 (41%) perforations healed completely, while 6 (14%) healed partially with at least 10% size reduction. In the control group, there were only 8 (22%) complete closures without any partial closures.

**Table 1.** Comparison of Patients’ Demographics and Baseline Perforation Characteristics Between Both Groups.

Characteristics	Experiment group (N = 44)	Control group (N = 37)	*P*-value
Age (years)	57 (51, 62)	59 (51, 66)	.60
Sex			.19
Female	21 (48%)	23 (62%)	
Male	23 (52%)	14 (38%)	
Race			.80
Asian	13 (30%)	12 (32%)	
Black or African American	1 (2%)	2 (6%)	
Other or Unknown	7 (16%)	4 (11%)	
White	23 (52%)	19 (51%)	
Hispanic ethnicity	11 (25%)	5 (14%)	.30
TM perforation etiology			.50
Trauma (baro, acoustic, direct)	6 (14%)	7 (19%)	
Otitis media	38 (86%)	30 (81%)	
Comorbidities			
Smoking	8 (18%)	7 (19%)	.90
Diabetes mellitus	10 (23%)	7 (19%)	.60
Location of perforation			.06
Mid-Inferior	10 (23%)	7 (19%)	
Anterior inferior	8 (18%)	4 (11%)	
Posterior inferior	10 (23%)	13 (35%)	
Anterior superior	4 (9%)	6 (16%)	
Posterior superior	6 (14%)	7 (19%)	
Mid-posterior	6 (14%)	0	
Size of perforation			.06
1%-10%	17 (39%)	25 (68%)	
11%-25%	13 (30%)	8 (22%)	
26%-40%	11 (25%)	2 (6%)	
41%-50%	2 (4%)	1 (3%)	
>50%	1 (2%)	1 (3%)	
Outcomes after the procedure			
Thickened TM	1 (2%)	4 (11%)	.10
TM atrophy	1 (2%)	0	.20
Tympanosclerosis	2 (4%)	2 (6%)	.80
TM retraction	5 (11%)	3 (8%)	.60
Granulation tissue	4 (9%)	1 (3%)	.20

**Figure 1.**
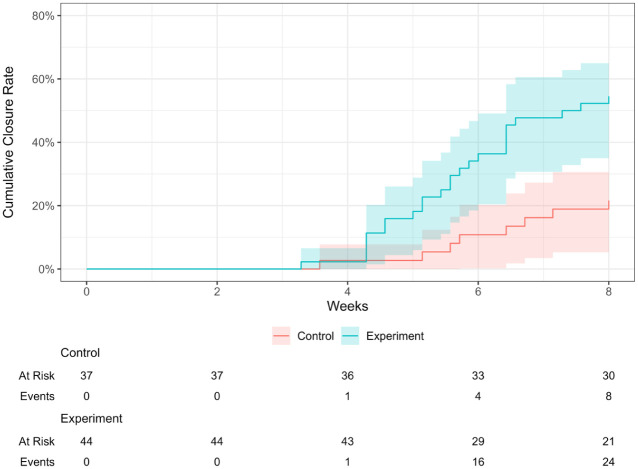
Kaplan-Meier survival curve for TM perforation closure. *Note.* Cumulative TM perforation closure over 8 weeks for the experimental (blue) and control (red) groups, with shaded 95% confidence intervals.

To conduct a more comprehensive survival analysis, partial closures in the experimental group were included along with complete closures. Kaplan-Meier analysis showed a significantly higher TM closure rate (χ^2^ = 9.6, *P* = .002) in the experimental group (55%, 95% CI: 37.2%-67.1%) compared to the control (22%, 95% CI: 7.2%-33.8%). Observed closures were 24 versus 8 in the experiment and control groups, respectively (Figure 1). The Cox proportional hazards model showed that age was not a significant predictor of TM closure over 8 weeks (HR = 0.995, 95% CI: 0.97–1.01, *P* = .60). TM closure rates did not significantly differ between females (40.9%, 95% CI: 24.4%-53.8%) and males (37.8%, 95% CI: 20.1%-51.7%; χ^2^ = 0.03, *P* = .85). Observed versus expected closures were similar (18 vs 17.5 in females; 14 vs 14.5 in males), indicating no association between sex and healing outcomes.

As shown in Table 2, TM closure rates did not significantly differ across patient demographics, perforation characteristics, or comorbidities. However, closure rates were higher for mid-posterior (67%) and posterior superior (61%) perforations compared to mid-inferior (23%) and posterior-inferior (30%) locations (*P* = .160). Smaller perforations (1%-10% and 11%-25%) had significantly higher healing rates, at 38% and 48%, respectively. The data on TM atrophy and granulation tissue were insufficient for conducting a survival analysis. The small number of observed events resulted in unstable estimates, wide confidence intervals, and reduced statistical power, making it unreliable to draw meaningful conclusions. Due to these limitations, any survival estimates would likely have been highly variable and prone to bias, hindering a thorough analysis of time-to-event outcomes for these conditions.

**Table 2.** Kaplan-Meier Estimates of Healing Rate at 8 Weeks After Procedure.

Variable	Cumulative incidence (%)	Lower CI (%)	Upper CI (%)	*P*-value
Group				.002
Control	21.6	7.2	33.8	
Experiment	54.5	37.2	67.1	
Sex				.85
Female	40.9	24.4	53.8	
Male	37.8	20.1	51.7	
Race				.57
Black	33.3	0.0	70.0	
Asian	44.0	20.7	60.4	
Other	18.2	0.0	38.1	
White	42.9	25.7	56.0	
Hispanic				.19
No	37.1	23.8	48.0	
Yes	56.2	23.7	74.9	
Etiology of perforation				.80
Otitis media	39.7	26.9	50.3	
Trauma	38.5	5.4	60.0	
Diabetes mellitus				.15
No	35.9	23.0	46.7	
Yes	52.9	22.1	71.6	
Smoking				.93
No	39.4	26.4	50.1	
Yes	40.0	9.3	60.3	
Laterality				.33
Left	45.7	26.4	59.9	
Right	34.8	19.5	47.2	
Location				.16
Anterior inferior	41.7	5.9	63.8	
Anterior superior	40.0	0.5	63.8	
Mid-inferior	23.5	0.5	41.3	
Mid-posterior	66.7	0.0	89.2	
Posterior inferior	30.4	8.8	46.9	
Posterior superior	61.5	23.5	80.7	
Baseline perforation size				.85
1%-10%	38.1	21.5	51.2	
11%-25%	47.6	21.2	65.2	
26%-40%	38.5	5.4	60.0	
41%-50%	33.3	0.0	70.0	
>50%	0.0	0.0	0.0	
Thickened TM				.44
No	40.8	28.6	50.9	
Yes	20.0	0.0	48.4	
Tympanosclerosis				.95
No	39.7	27.8	49.7	
Yes	33.3	0.0	70.0	
Retraction				.30
No	38.4	26.1	48.6	
Yes	50.0	0.0	75.0	

## Discussion

The results of this study indicate that adding autologous serum drops to paper patching significantly enhances the success rate of TM perforation closure. Kaplan-Meier survival analysis showed that the cumulative incidence of TM healing was significantly higher in the experimental group (55%) compared to the control group (22%), with a statistically significant *P*-value of .002. These findings indicated that serum drops play a beneficial role in enhancing TM repair, supporting previous studies investigating the potential of bioactive substances in TM regeneration.^17,18^

In their study, Kakehata et al^17^ evaluated a non-surgical, office-based approach for chronic TM perforations using silver nitrate cauterization, a chitin membrane scaffold, and autologous serum eardrops, achieving a 58% closure rate. While a small subgroup treated with cauterization and chitin membrane alone served as a quasi-control, the lack of a formal control group remained a methodological limitation. Consequently, they suggested that further research is necessary to verify the specific contribution of autologous serum drops. When considered alongside the findings of this study, it seems that the application of adjunct autologous serum drops promotes TM regeneration. In another study, the impact of autologous serum drops on TM healing in rat models with traumatic perforation was investigated.^18^ The authors attempted an intra-subject comparison using 20 rats and inflicting trauma to both TMs within the same relative period; 1 TM served as a control, and the other was treated with autologous serum drops.^18^ Two key aspects of this study were worth noting: the acute nature of the trauma and the relatively small sample size. They induced TM trauma acutely, which generally heals spontaneously at a high rate.^16,18^ The results reflected this, as 17 out of 20 in the control group had completely closed their TM. Since acute TM perforation tends to heal, the results may not accurately represent conditions involving chronic TM perforation. The high spontaneous healing rate (85%) in the control group indicates the study by Karaman et al^18^ was not powered enough to detect a difference between the experimental group and the control.

Lou et al studied the effects of ofloxacin drops on acute TM regeneration.^22^ The study found that patients with medium-sized perforations (less than 25% of TM surface area) treated with ofloxacin drops had shorter closure times than those in the observation group.^22^ However, there was no significant difference in closure rates between the 2 groups. In large perforations (over 25% of the TM), the ofloxacin-treated group showed improved closure rates and reduced mean closure time.^22^ They reported improved closure rates of TM, attributing the benefit to a moist healing environment; however, the authors did not clarify whether the observed effect was due to the antibiotic properties of ofloxacin or its hydrating role, limiting the interpretability of their findings. They lacked a control group receiving saline ear drops, which would have helped distinguish the specific contribution of moisture to the healing process. In addition, ofloxacin does not possess any known regenerative or growth-promoting properties beyond its antimicrobial action.

Our study found that the anatomical location of TM perforations affected healing outcomes. Posterior and posterior superior perforations showed relatively higher closure rates compared to inferior and posterior-inferior perforations (Table 2). Although it did not reach statistical significance (*P* = .16). The influence of perforation location TM healing remains unclear. Although our finding of improved healing in mid posterior and posterior superior perforations is notable, it may be confounded by etiology, as these perforations were typically dry and post-myringotomy, whereas mid-inferior and posterior inferior ones were often infection-related. Superior regions may benefit from better vascular supply or more favorable anatomy for patch adherence and epithelial migration, while inferior sites may be disadvantaged by poorer vascularization or middle ear effusions. Further research is needed to determine whether location itself promotes healing or reflects underlying factors such as Eustachian tube function or infection.

The size of TM perforations is also a crucial factor in determining healing potential. Our results showed that larger perforations (41%-50% and >50%) exhibited lower healing rates in the experimental group versus the control (33% vs 0%), whereas smaller perforations (0%-10% and 11%-25%) showed a higher closure rate (38% and 48%, respectively). Multiple studies have indicated that both spontaneous and procedural healing were enhanced in smaller perforations, attributed to the reduced migration distance required for epithelial and fibrous tissue.^4,23^ Larger perforations may experience greater exposure to environmental irritants and inadequate epithelial bridging, leading to a reduced likelihood of spontaneous closure.^23^

Since it is a controlled study that focuses on patients who had failed paper patching, any observed benefits or adverse effects experienced by the patients are likely attributable to the application of adjuvant autologous serum drops. Autologous serum drops represent a promising adjunct to paper patch placement for chronic TM perforations. Traditionally, the treatment of chronic TM perforations relied on standard procedures like myringoplasty or tympanoplasty. While effective, these procedures are associated with challenges, including high costs, the need for specialized surgical expertise, and the risk of surgery and postoperative complications.^6^ In response to these limitations, cost-effective outpatient alternatives such as paper-patch myringoplasty and the use of autologous serum drops have been explored as viable solutions.^6,24^ Their effectiveness stems from their rich composition of biomolecules linked to tissue regeneration and wound healing, including glucose, electrolytes, VEGF, lymphocytes, and collagen fibrils.^17,18,20,21^ It also contains hyaluronic acid, as well as fibroblast growth factors (FGF), all of which have been previously recognized independently for promoting TM regeneration.^19,20,25,26^ Hyaluronic acid forms a thin keratin film that allows proper epithelial cell migration for perforation closure and regulates collagen deposition to prevent fibrosis.^10,16^ Our study posits that tissue or organ regeneration needs sufficient scaffolding, regulatory factors, and cellular activity. Chronic TM perforations may result from a deficiency of these components, hindering adequate healing and chronicity.^10,15,16^ Therefore, supplementing serum drops may address the lack of regenerative components and promote TM regeneration.

A different theory has been suggested about chronic TM perforation, indicating that the condition persists due to the re-epithelialization of the perforation margin. According to this theory, the re-epithelialized membrane is viewed as healed by the body, even if complete regeneration has not occurred.^25,27^ In our study, we freshened the perforation margins before application of the paper patch. This action aimed to make the perforation more similar to acute rather than chronic one.^25,27^ However, it has been found that disrupting the re-epithelialized edges is inadequate for the complete healing of chronic TM perforations.^27^ These perforations failed to respond to a paper patch without serum drops, so we believe that those who had closure benefited from the serum drops. It is important to note that autologous serum is derived from the patient’s own blood, thereby lacking antigenicity.^19-21^ Its use was definitely ensured not to trigger hypersensitivity reactions, as no adverse effects or allergies were reported during the follow-up period.

Although this study aimed to isolate the effects of autologous serum drop application on TM regeneration, several limitations and considerations are worth noting. Its retrospective design introduced a lack of randomization and possible selection bias. Although the baseline demographic variables, perforation characteristics, and comorbidities were not statistically different between the experimental and control groups, minimizing the likelihood of systematic group imbalance, unmeasured confounding factors may still have influenced treatment choice or healing outcomes. The relatively small sample size, particularly within subgroups, limited statistical power and precision, especially for less common variables such as large perforations or TM atrophy. The follow-up period was limited to 8 weeks, preventing assessment of long-term repair durability or late complications such as re-perforation or scarring. Additionally, treatment adherence to serum drop protocols was not objectively monitored, and the absence of blinding may have introduced performance or assessment bias. These limitations underscore the need for larger, prospective studies with longer follow-up to validate and expand upon these findings.

## Conclusions

This study highlights the benefits of supplementing paper patching with autologous serum drops as an affordable, non-surgical method to improve TM perforation closure rates, influenced by perforation size and location. Future research should examine larger sample sizes and long-term efficacy.
